# The Anatomy and Physiology of the Nervous System and Its Constituent Neurones, as Revealed by Recent Investigations

**Published:** 1900-07

**Authors:** 


THE ANATOMY AND PHYSIOLOGY OF THE NERVOUS SYSTEM AND ITS CONSTITUENT NEURONES, AS REVEALED BY RECENT INVESTIGATIONS. Designed for the use of practitioners of medicine and students of medicine and psychology. By Lewellys F. Barker, M. B., Associate Professor of Anatomy in the Johns Hopkins University and Assistant Resident Pathologist to the Johns Hopkins Hospital. 8 vo, with over 800 illustrations. Sold by subscription. Cloth, $5. New York: D. Appleton & Co. 1900.

The first part of this volume is devoted to a discussion of the newer conceptions of the histology of the central and peripheral nervous system. The doctrine of cell, or neurone, conception is applied in explanation of the complex architectonics of the nervous system, the term *neurone* being used in its widest sense to mean "a cell belonging to the nervous system with all its parts." The book is of great importance at this time as it discusses the newer ideas of nervous action, giving minutely the anatomy and physiology, together with much original work. Every student who delves deeply into subjects, this book will lead into new fields of facts and inspiration. T. J. B.

